# Mapping proteins in the presence of paralogs using units of coevolution

**DOI:** 10.1186/1471-2105-14-S15-S18

**Published:** 2013-10-15

**Authors:** Mohammed El-Kebir, Tobias Marschall, Inken Wohlers, Murray Patterson, Jaap Heringa, Alexander Schönhuth, Gunnar W Klau

**Affiliations:** 1 Centrum Wiskunde & Informatica (CWI), Life Sciences Group, Amsterdam, The Netherlands; 2 Centre for Integrative Bioinformatics VU, VU University Amsterdam, The Netherlands; 3 University of Duisburg-Essen, Genome Informatics, Essen, Germany

## Abstract

**Background:**

We study the problem of mapping proteins between two protein families in the presence of paralogs. This problem occurs as a difficult subproblem in coevolution-based computational approaches for protein-protein interaction prediction.

**Results:**

Similar to prior approaches, our method is based on the idea that coevolution implies equal rates of sequence evolution among the interacting proteins, and we provide a first attempt to quantify this notion in a formal statistical manner. We call the units that are central to this quantification scheme the *units of coevolution*. A unit consists of two mapped protein pairs and its score quantifies the coevolution of the pairs. This quantification allows us to provide a maximum likelihood formulation of the paralog mapping problem and to cast it into a binary quadratic programming formulation.

**Conclusion:**

CUPID, our software tool based on a Lagrangian relaxation of this formulation, makes it, for the first time, possible to compute state-of-the-art quality pairings in a few minutes of runtime. In summary, we suggest a novel alternative to the earlier available approaches, which is statistically sound and computationally feasible.

## Introduction

Protein-protein interactions are essential for understanding cellular mechanisms and their malfunctioning in disease [1]. Both experimental and computational methods exist for their prediction [2]. Among the latter, many are based on the observation that interacting proteins often have coevolved due to a positive selection pressure on preserving the interaction [3-6]. This observation allows to predict protein-protein interactions by quantifying the degree of similarity between the evolution of two protein families. Coevolution-based methods map proteins across the families in order to maximize a similarity measure between the phylogenetic trees or the underlying distance matrices. In settings with only orthologous proteins (e.g. [7], a study on coevolution in prokaryotes), the mapping task is trivial as every protein family contains only one protein per species. In the presence of paralogous proteins (paralogs), however, the mapping task becomes difficult.

There are only a handful of existing approaches for the *paralog mapping problem *[8-10]. Izarzugaza et al. [8], in their method TAG-TSEMA, and most earlier approaches establish mappings by swapping rows and columns of the distance matrices to achieve similarity between the matrices. Tillier et al. [9] take a different approach in their method MMM by heuristically determining submatrices of the two distance matrices to be paired. The recent approach TreeTop by Hajirasouliha et al. [10] computes mappings by comparing two phylogenetic trees derived from the multiple sequence alignments using dynamic programming. Compared to the matrix-based method [8] this yields a speed-up of several orders of magnitude, which, however, comes at the expense of significantly reduced, incomplete mappings.

Here, we present a new mathematical model and method, which are based on statistically quantifying the degree of coevolution reflected by a mapping. Similar to prior approaches, our method is based on the idea that coevolution implies equal rates of sequence evolution among the interacting proteins, and we provide a first attempt to quantify this notion in a formal statistical manner. We call the units that are central to this quantification scheme the *units of coevolution*. A unit consists of two mapped protein pairs and its score quantifies the coevolution of the pairs. The quality of a mapping is then rated in terms of the units of coevolution it consists of. We establish and exploit a connection to the global network alignment problem and are thus able to find provably near-optimal or optimal mappings. Due to the design of our quality scores, an optimal mapping corresponds to a maximum likelihood estimate of a generative statistical model built upon the participating units of coevolution. We extend a recent Lagrangian relaxation approach for network alignment [11] to deal with the new scoring scheme. We apply our method to an approved benchmark of coevolving protein domains. In terms of recall and precision, we outperform MMM, perform better than TreeTop and slightly better than TAG-TSEMA. In terms of runtime, we outperform TAG-TSEMA by an order of magnitude, are faster than MMM and much slower than TreeTop.

Our software tool CUPID (Coevolution Units Paralog Interaction Detector) as well as all data and scripts to reproduce the results are freely available as part of the NINA project for network analysis and integration at http://www.cwi.nl/research/nina.

## Mathematical model

### Units of coevolution

**Figure 1:**
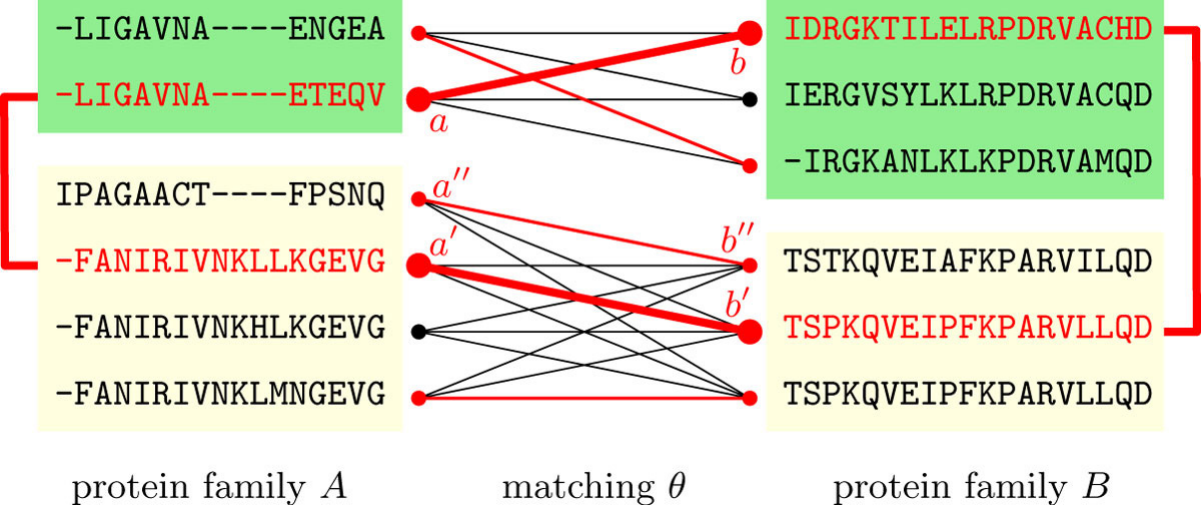
**Two alignments of protein families *A *and *B *with proteins from two species, which are indicated by different background colors**. Black and red nodes and edges compose the matching graph *G*. A matching *θ *is shown in red. A unit of coevolution ((*a, b*), (*a*'*, b*')) within *θ *is highlighted in bold. For this toy example, we have *ℓ_A_*(*a, a*') = 12 (matches + mismatches), ∆*_A_*(*a, a*') = 11 (mismatches), *ℓ_B _*(*b, b*') = 19 and ∆*_B _*(*b, b*') = 15 and a resulting probability $f\left({\text{Δ}}_{A}\left(a,a^{\prime }\right),{\text{Δ}}_{B}\left(b,b^{\prime }\right)\right)=\left(\begin{array}{c} 12\\ 11 \end{array}\right)\left(\begin{array}{c} 19\\ 15 \end{array}\right)/\left(\begin{array}{c} 31\\ 26 \end{array}\right)\approx 0.274$. Note the lower score of the unit ((*a*'*, b*'), (*a*"*, b*")), which is $\left(\begin{array}{c} 12\\ 11 \end{array}\right)\left(\begin{array}{c} 19\\ 3 \end{array}\right)/\;)\left(\begin{array}{c} 31\\ 14 \end{array}\right)\approx 4.4\cdot 10^{-5}$.

The data we take as input are multiple alignments of two supposedly interacting protein families. In line with previous work [8-10,12], we assess coevolution in terms of the differences of sequence identities derived from the multiple alignments. Here we stick to earlier practice and define sequence identity as the number of mismatches divided by the sum of matches and mismatches without counting gap columns. Given sets of sequences *A *and *B *representing the two supposedly interacting families whose members are to be paired, let a* and b* be common ancestral sequences of *A *and *B*, respectively. Now, we look for pairs (*a, b*) ∈ *A × B *such that the sequence identity between *a *and a* equals the sequence identity between *b *and b*. The caveat here, however, is that a* and b* are unknown. Hence, we cannot infer the degree of coevolution of two family members *a *∈ *A *and *b *∈ *B *by considering the pair (*a, b*) alone. To overcome this, we consider quadruples, i.e., pairs of pairs (*a, b*) and (*a*', *b*'), and assess them based on the following idea: if *a *and *a*' are significantly more similar to each other than *b *is to *b*', or vice versa, then at least one of the pairs (*a, b*), (*a*', *b*') is likely to represent non-coevolving proteins. This is because the differences in sequence identity among each other imply different rates of divergence from the virtual, common ancestors a* and b*. Using a* and b* instead of the two most recent common ancestors is justified by the common assumption that the trees of interacting protein families are near identical [8,10]. We call quadruples ((*a, b*), (*a*'*, b*')) *units of coevolution*. The main theme of this paper is to determine a matching (i.e., a mapping) of family members that is optimal with respect to the quadruples it contains. See Figure 1 for an illustration and the next subsection for how to assign statistically motivated values to units of coevolution.

### Maximum likelihood maximum cardinality matchings

In the following, we provide a formal definition of *units of coevolution*. Based on this, we develop a statistical model that can be interpreted as generating units of coevolution and that is parameterized by matchings. Determining an optimal matching then translates to determining the maximum likelihood estimate of the observed data. To do this, we need the following notation:

**Definition 1 (Matching graph) ***Let A and B be protein families whose members v *∈ *A *∪ *B are labeled by their species s*(*v*)*. The *matching graph *is a bipartite graph G *= (*A *∪ *B, E*) *where E *= {(*a, b*) ∈ *A × B | s*(*a*) = *s*(*b*)}.

A *matching *of *G *is a subset of edges such that no two edges are incident to the same node. When *S *is the set of all species, the mapping *s *: *A *∪ *B *→ *S *used above induces partitions of *A *and *B*. We define *A_t _*:= {*a *∈ *A | s*(*a*) = *t*} and *B_t _*:= {*b *∈ *B *| *s*(*b*) = *t*} to refer to the respective parts of species *t*. Because *G *consists of |*S*| connected components, which are complete bipartite subgraphs, all maximal matchings of *G *have the same cardinality

$$n=\underset{t\in S}{\sum }\text{min}\left\{\left|A_{t}|,\;|B_{t}\right|\right\}.$$

Now, we define our search space as follows.

**Definition 2 (Search space) ***The *search space Θ *is the set of matchings of maximum cardinality n*.

Next, we develop a parametrized statistical model whose parameters can be identified with the search space Θ. As pointed out above, a maximum likelihood estimate θ* ∈ Θ then corresponds to an optimal matching and hence an optimal pairing of putatively coevolving family members. Let ∆*_A_*(*a, a*') be the number of sequence mismatches between *a *and *a*' and let *ℓ_A_*(*a, a*') be the number of sequence matches and mismatches between *a *and *a*' in the multiple alignment *A*. See Figure 1 for an example.

We make two simplifying assumptions to derive a suitable problem formulation. First, we assume a hidden substitution rate *p_a, a' _*for each pair of sequences *a, a*' ∈ *A *such that the observed quantity of ∆*_A_*(*a, a*') follows a binomial distribution with parameter *p_a, a'_*. That is, we model mismatches by independent Bernoulli trials with probability *p_a, a'_*. We make the analogous assumption for all *b, b*' ∈ *B*. Therefore, if *a *interacts with *b *and *a*' with *b*', observing numbers ∆*_A_*(*a, a*') and ∆*_B _*(*b, b*') together is described by a hypergeometric distribution. Formally, the probability for observing ∆*_A_*(*a, a*') and ∆*_B _*(*b, b*') given *ℓ_A_*(*a, a*'), *ℓ_B _*(*b, b*'), and ∆*_A_*(*a, a*') + ∆*_B _*(*b, b*') is given by

(1) $$\begin{array}{cc} f\left({\text{Δ}}_{A}\left(a,\;a^{\prime }\right),{\text{Δ}}_{B}\left(b,\;b^{\prime }\right)\right) & =P_{H}\left({\text{Δ}}_{A}\left(a,\;a^{\prime }\right),{\text{Δ}}_{B}\left(b,\;b^{\prime }\right)|\ell_{A}\left(a,\;a^{\prime }\right),\ell_{B}\left(b,\;b^{\prime }\right),{\text{Δ}}_{A}\left(a,\;a^{\prime }\right)+{\text{Δ}}_{B}\left(b,\;b^{\prime }\right)\right)\\ & =\frac{\left(\begin{array}{c} \ell_{A}\left(a,\;a^{\prime }\right)\\ {\text{Δ}}_{A}\left(a,\;a^{\prime }\right) \end{array}\right)\left(\begin{array}{c} \ell_{B}\left(b,\;b^{\prime }\right)\\ {\text{Δ}}_{B}\left(b,\;b^{\prime }\right) \end{array}\right)}{\left(\begin{array}{c} \ell_{A}\left(a,\;a^{\prime }\right)+\ell_{B}\left(b,\;b^{\prime }\right)\\ {\text{Δ}}_{A}\left(a,\;a^{\prime }\right)+{\text{Δ}}_{B}\left(b,\;b^{\prime }\right) \end{array}\right)}, \end{array}$$

where *H *is the assumption of equal evolutionary rates due to coevolution.

**Definition 3 (Unit of coevolution) ***We refer to *(1) *as the value of the unit of coevolution *((*a, b*), (*a*'*, b*')).

We now assume that all units of coevolution are independent. The overall likelihood of a matching *θ *is thus

(2) $$f\left({\text{Δ}}_{A},{\text{Δ}}_{B};\theta \right)=\underset{\begin{array}{c} \left(a,b\right)\left(a^{\prime },b^{\prime }\right)\in \theta \\ \left(a,b\right)<\left(a^{\prime },b^{\prime }\right) \end{array}}{\prod }f\left({\text{Δ}}_{A}\left(a,\;a^{\prime }\right),{\text{Δ}}_{B}\left(b,\;b^{\prime }\right)\right),$$

where "<" is an arbitrary ordering on *E*.

The independence assumption may, at first glance, appear unjustified because a pair (*a, b*) can take part in many units of coevolution. Note, however, first that it is equivalent to maximize $\sqrt[{\left(n-1\right)/2}]{f\left({\text{Δ}}_{A},{\text{Δ}}_{B};\theta \right)}$ instead of (2) where *n *is the size of the matching *θ*. Rewriting

$$\sqrt[{\left(n-1\right)/2}]{f\left({\text{Δ}}_{A},{\text{Δ}}_{B};\theta \right)}=\underset{\left(a,b\right)\in \theta }{\prod }C\left(a,b;\theta \right)$$

where

$$C\left(a,b;\theta \right):=\sqrt[{n-1}]{\underset{\left(a^{\prime },b^{\prime }\right)\in \theta ,\left(a^{\prime },b^{\prime }\right)\neq \left(a,b\right)}{\prod }f\left({\text{Δ}}_{A}\left(a,a^{\prime }\right),{\text{Δ}}_{B}\left(b,b^{\prime }\right)\right)}$$

which one can -- as the (harmonic) mean of all units of coevolution (*a, b*) takes part in -- interpret as a measure for the degree of coevolution of the individual pair (*a, b*). It is now reasonable to believe that the degrees of coevolution of (*a, b*) and (*a*'*, b*') are independent of one another: This clearly applies if the two pairs stem from two different species (that is, *a *is orthologous to *a*' and *b *is orthologous to *b*'), because there is usually no genetic crosstalk across species, at least not in eukaryotes. Even in the case of *a *being paralogous to *a*' and *b *being paralogous to *b*', the assumption of independence may be reasonable, because paralogs often assume functions that considerably diverge from their paralogous partners, hence are subject to independent selective pressures. So, one can decompose (2) into factors, for which the assumption of independency makes sense, while each factor has a reasonable interpretation. This may justify the assumption of independency overall.

The problem is now as follows.

**Problem 1 (Maximum likelihood maximum cardinality matching) ***Let A and B be two protein families whose proteins v *∈ *A *∪ *B are labeled by their species s*(*v*), *let G be the corresponding bipartite graph and let *Θ *be the set of maximum cardinality matchings as given in Definitions 1 and 2, respectively*. *Then, the goal is to find the maximum likelihood matching*

$$\theta^{*}=\underset{\theta \in \Theta }{\text{arg }\text{max}}f\left({\text{Δ}}_{A},{\text{Δ}}_{B};\theta \right).$$

## Method

We start by formulating the problem as a binary quadratic program (BQP). For notational convenience, we switch from using *a, a*' ∈ *A *and *b, b*' ∈ *B *to using *i, j *∈ *A *and *k, l *∈ *B*. As a first step, we take the logarithm of (2), which yields the log likelihood

(3) $$\text{log}f\left({\text{Δ}}_{A},{\text{Δ}}_{B};\theta \right)=\underset{\begin{array}{c} \left(i,k\right),\left(j,l\right)\in \theta \\ \left(i,k\right)<\left(j,l\right) \end{array}}{\sum }\text{log}f\left({\text{Δ}}_{A}\left(i,\;j\right),{\text{Δ}}_{B}\left(k,l\right)\right).$$

We represent a matching *θ *by binary variables *x_ik _*which are equal to 1 if and only if the edge (*i, k*) is in *θ*. As a shorthand we use *f_ijkl _*= log *f *(∆*_A_*(*i, j*), ∆*_B _*(*k, l*)). Now the corresponding quadratic program is

$$\underset{x}{\text{max}}\underset{\begin{array}{c} i,j\\ i<j \end{array}}{\sum }\underset{\begin{array}{c} k,l\\ k\neq l \end{array}}{\sum }f_{ijkl}x_{ik}x_{jl}$$

(BQP-1)

(4) $$\text{s}\text{.t}\text{.}\;\underset{k}{\sum }x_{ik}\leq 1\;\;\;\forall i$$

(5) $$\underset{i}{\sum }x_{ik}\leq 1\;\;\;\forall k$$

(6) $$\underset{i,k}{\sum }x_{ik}=n$$

(7) $$x_{ik}=0\;\;\;\forall i,\;k,\;s\left(i\right)\neq s\left(k\right)$$

(8) $$x_{ik}\in \left\{0,1\right\}\;\;\;\forall i,\;k$$

Constraints (4) and (5) are the standard constraints for bipartite matching. Equality (6) ensures that the matching will have maximum cardinality. Constraints (7) ensure that only proteins of the same species are mapped. The quadratic objective function scores the contribution of units of coevolution, which may consist of protein pairs that belong to different species. We formally show how to transform this integer linear programming formulation into a well-studied formulation used for the Quadratic Assignment Problem [13] and for network alignment [11,14].

To this end, we eliminate constraint (6) by shifting all *f_ijkl _*by an offset *K *> 0 such that they become strictly positive. Correcting for this in the objective function leads to

$$\underset{x}{\text{max}}\underset{\begin{array}{c} i,j\\ i<j \end{array}}{\sum }\underset{\begin{array}{c} k,l\\ k\neq l \end{array}}{\sum }\left(f_{ijkl}+K\right)x_{ik}x_{jl}-\left(\begin{array}{c} n\\ 2 \end{array}\right)\cdot K\;\;\text{s}\text{.t}\text{.}\;\text{(4),}\;\text{(5),}\;\text{(7)}\;\text{and}\;\text{(8)}\text{.}$$

(BQP-2)

(BQP-1) and (BQP-2) are the same as shown in the following lemma.

**Lemma 1 ***A solution θ *∈ Θ *is optimal to *(BQP-1) *if and only if it is optimal to *(BQP-2)*. Furthermore, the objective value of θ in *(BQP-1) *is equal to the objective value of θ in *(BQP-2).

*Proof*. Let *θ*_1 _be an optimal solution to (BQP-1) and *θ*_2 _an optimal solution to (BQP-2). Let *G *= (*A *∪ *B, E*) be the matching graph as introduced in Def. 1.

We start by showing that |*θ*_1_| = |*θ*_2_| = *n*. By constraint (6), we have that |*θ*_1_| = *n*. To prove |*θ*_2_| = *n*, we recall that *G *consists of connected components induced by *A_t _*∪ *B_t _*for *t *∈ *S*, each of which is a complete bipartite subgraph. Suppose that *θ*_2 _is not maximal, i.e., |*θ*_2_| <*n*. Observe that every component *A_t _*∪ *B_t _*can have at most min{|*A_t_*|, |*B_t_*|} matched nodes in *θ*_2_. As $n=\sum_{t\in S}\text{min}\left\{\left|A_{t}|,|B_{t}\right|\right\}$ and |*θ*_2_| <*n*, there must exist a component *t *with unmatched nodes *a *∈ *A_t _*and *b *∈ *B_t_*. Since *f_ijkl _*+ *K *> 0 for all quadruples ((*i, j*), (*k, l*)) with *i *<*j *and *k *≠ *l*, we have that *θ*_2 _is not an optimal solution for (BQP-2) as including (*a, b*) in the matching would result in a matching with a greater objective value. Therefore, it follows that |*θ*_1_| = |*θ*_2_| = *n*.

The number of quadruples, or units of coevolution, induced by any maximum cardinality matching is $\left(\begin{array}{c} n\\ 2 \end{array}\right)$. Therefore, any *maximum cardinality matching *that is a feasible solution to (BQP-1) and (BQP-2) has an objective value of

(9) $$\underset{\begin{array}{c} i,j\\ i<j \end{array}}{\sum }\underset{\begin{array}{c} k,l\\ k\neq l \end{array}}{\sum }\left(f_{ijkl}+K\right)x_{ik}x_{jl}-\left(\begin{array}{c} n\\ 2 \end{array}\right)\cdot K=\underset{\begin{array}{c} i,j\\ i<j \end{array}}{\sum }\underset{\begin{array}{c} k,l\\ k\neq l \end{array}}{\sum }f_{ijkl}x_{ik}x_{jl}.$$

As |*θ*_1_| = |*θ*_2_| = *n*, the above equality also holds for matchings *θ*_1 _and *θ*_2_. In addition, *θ*_1 _is by definition feasible to (BQP-2). Conversely, *θ*_2 _is feasible to (BQP-1) as |*θ*_2_| = *n*. Therefore, we have that optimal solutions to (BQP-1) and (BQP-2) have equal objective values.     QED

Our starting point for the Lagrangian relaxation is (BQP-2) where the weights assigned to the quadruples are strictly positive. We obtain the relaxation along the same lines as in [11]. The main resulting theorem is as follows.

**Theorem 1 ***Let *$m=\left(\underset{2}{{\text{∑}}_{\text{t∈s}}\text{|}{\text{A}}_{\text{t}}\text{|⋅|}{\text{B}}_{\text{t}}\text{|}}\right).$*For any *$\lambda \in {\mathbb{R}}^{m}$*, an *upper bound *on *(BQP-2) *is given by*

$$Z_{{\text{LD}}_{\left(\text{λ}\right)}}=\underset{x}{\text{max}}\underset{i,k}{\sum }v_{ik}\left(\text{λ}\right)\cdot x_{ik}$$

(LD*_λ_*)

(10) $$s.t.\;\underset{k}{\sum }x_{ik}\leq 1\;\;\;\forall i$$

(11) $$\underset{i}{\sum }x_{ik}\leq 1\;\;\;\forall k$$

(12) $$x_{ik}=0\;\;\;\forall i,k,s\left(i\right)\neq s\left(k\right)$$

(13) $$x_{ik}\in \left\{0,1\right\}\;\;\;\forall i,k$$

where

$$v_{ik}\left(\lambda \right)=\underset{y}{\text{max}}\underset{\begin{array}{c} j\\ j>i \end{array}}{\sum }\underset{\begin{array}{c} l\\ l\neq k \end{array}}{\sum }\left(w_{ijkl}+\lambda_{ijkl}\right)y_{ijkl}+\underset{\begin{array}{c} j\\ j<i \end{array}}{\sum }\underset{\begin{array}{c} l\\ l\neq k \end{array}}{\sum }\left(w_{ijkl}+\lambda_{ijkl}\right)y_{ijkl}\;\;\;\;\left({\text{LD}}_{\lambda }^{ik}\right)$$

(14) $$s.t.\;\underset{\begin{array}{c} l\\ l\neq k \end{array}}{\sum }y_{ijkl}\leq 1\;\;\;\;\;\forall j,\;\;j\neq i$$

(15) $$\underset{\begin{array}{c} j\\ j\neq 1 \end{array}}{\sum }y_{ijkl}\leq 1\;\;\;\;\;\forall l,\;\;l\neq k$$

(16) $$y_{ijkl}\in \left\{0,1\right\}\;\;\;\;\forall j,l$$

*and where w_ijkl _*= (*f_ijkl _*+ *K*)/2. *The upper bound Z*_LD_(*λ*) *can be computed in time  O*(*n*^5^).

In the theorem above each variable *y_ijkl _*refers to a unit of coevolution. Since (BQP-2) is the formulation used for global network alignment in [14] and [11], upper bound and runtime follow directly from the proof given in [11]. We obtain solutions to (LD*_λ_*) and $\left({\text{LD}}_{\lambda }^{ik}\right)$ by solving the corresponding maximum weight bipartite matching problems. From a solution (*x, y*) to (LD*_λ_*), we compute a feasible solution to (BQP-2) by using the matching encoded in *x *whose score is a lower bound on the value of the optimal solution to (BQP-2). The goal now is to identify λ* which results in the smallest gap between upper and lower bound. We do this using a hybrid procedure combining subgradient optimization and a specially crafted dual descent scheme. For details we refer again to [11].

## Results

### Benchmark data set

Designing a large benchmark data set for our problem is difficult as there is insufficient information on the interaction between the individual members of protein families and the correct mapping of paralogs is thus usually unknown. We therefore rely on the reference data set of Izarzugaza et al. [8] in which the protein families are in fact domain families and the type of interaction is the co-occurrence in the same protein chain. The task is to determine a correct matching between protein domains of the same species. In this benchmark, a correct matching maps only domains that occur in the same protein chain and are therefore known to coevolve. Izarzugaza et al. [8] compiled the data set by first selecting Pfam [15] domains that co-occur in known yeast proteins and then took from these domains all eukaryotic sequences present in SwissProt which are not labeled "fragment", "hypothetic" or "putative". Finally they selected those domain pairs which (i) per family cover at least four species with at least three sequences each, (ii) in which at least 15 sequences are mapped, i.e., co-occur in a protein chain, and (iii) which have at least 50% of the sequences of the domain with fewest members mapped. The resulting benchmark instances comprise 488 pairs of multiple sequence alignments of domain families whose domains co-occur in the same protein chain. The total number of domain families in the benchmark is 604 and the number of domains per domain family ranges from 21 up to 212.

In previous work, phylogenetic trees were constructed from the alignments and either the trees themselves [10] or the distance matrices derived from them [8,9] were compared. In contrast, our algorithm uses data from the multiple alignments directly for scoring, as detailed in the Mathematical Model section. In addition to the alignments, the species from which each sequence originates is provided as input to the algorithms. We ran the experiments for CUPID and MMM on a 2.26 GHz processor with 24 GB of RAM, running 64-bit Linux. For MMM we vary the allowance parameter *a *between 0.1 and 0.5. For TAG-TSEMA and TreeTop we took the numbers from [10]. Note that TAG-TSEMA was run on one of the fastest supercomputers at the time (2007/8). TreeTop was run on a similar machine as used for CUPID.

### Recall and precision

For each instance, we compute the recall and precision of the predicted matching with respect to the reference solution, which is the largest matching in which only domains of the same protein are paired, i.e., domains that are known to coevolve. Recall is defined as the percentage of correctly predicted pairings with respect to the cardinality of the reference solution. Precision is defined as the number of correctly predicted pairings divided by the cardinality of the predicted solution.

### Solution quality and runtime

Table 1 lists recall and precision for TAG-TSEMA [8], TreeTop [10], MMM [9], and CUPID. For MMM we applied a wall-time limit of 1 hour per instance. The number of instances that MMM could solve within the time limit rapidly decreases with increasing *a*. Our method CUPID achieves a recall of 56 % and a precision of 50 %, improving on the other methods. Also in comparison with MMM, CUPID achieves higher recall and precision on the subset of instances that were solved by MMM for varying values of *a*. Further, CUPID outperforms TAG-TSEMA by an order of magnitude in terms of runtime. TreeTop is much faster than CUPID (0.02 h as compared to 30 h) at the expense of a substantially worse recall (38 % compared to 56 %).

**Table 1:** The average recall and precision values in percent as well as the runtime in hours of TAG-TSEMA [8], TreeTop [10], MMM [9] and our method CUPID are shown.

	Recall	Precision	Runtime	#Instances
TAG-TSEMA [8]	**56 %**	45 %	730 h	488
TreeTop [10]	38 %	48 %	**0.02 h**	488
CUPID	**56 %**	**50 %**	30 h	488
MMM, *a *= 0.1 [9]	6 %	35 %	55 h	488
MMM, *a *= 0.2 [9]	15 % [61 %]	46 % [55 %]	121 h	394
MMM, *a *= 0.3 [9]	26 % [70 %]	57 % [64 %]	250 h	270
MMM, *a *= 0.4 [9]	35 % [71 %]	53 % [65 %]	323 h	214
MMM, *a *= 0.5 [9]	37 % [70 %]	44 % [65 %]	363 h	149

CUPID was terminated when either optimality was reached or a time limit of 5 minutes was hit; in the latter case, the best solution found until that time was used. TAG-TSEMA and TreeTop values are taken from [10]. MMM runs were subject to a time limit of 1 hour; the number of instances solved within this time limit are given in the last column. Precision and recall values are only determined for the set of solved instances. For the same set of solved instances the CUPID quality measure is given in square brackets.

CUPID terminates if either a maximum runtime is reached or the optimal solution has been found. If the time limit is hit, it returns a feasible solution and an upper bound on the optimal score. By definition, the score of the returned solution is a lower bound on the optimal score. We define the *relative gap *as the difference between upper and lower bound relative to the absolute value of the lower bound. To determine a good maximum runtime, we ran CUPID on all instances with maximum single-CPU-core runtimes of 10 sec, 30 sec, 1 min, 5 min, 10 min, and 20 min. Table 2 summarizes the effect on solution quality in terms of precision, recall, median relative gap size, and the number of instances solved to optimality. These results confirm that precision and recall increase with maximum runtime, while the median relative gap size decreases. This converging behavior suggests that our scoring function correlates well with precision and recall and that our algorithm is robust with respect to the choice of the time limit. Based on Table 2, we decided that stopping after 5 min represents a good trade-off between runtime and solution quality. By increasing the runtime from 5 min to 20 min, recall and precision both increase only by less than one percentage point. On the other hand, going from 5 min to 1 min, recall and precision both drop by more than 1.4 percentage points.

**Table 2:** Effect of time limit on solution quality of CUPID.

Time limit	10 sec	30 sec	1 min	5 min	10 min	20 min
Total runtime	1.3 h	3.8 h	7.3 h	30.2 h	51.6 h	81.0 h
Precision	46.8 %	47.8 %	48.2 %	49.6 %	49.8 %	50.3 %
Recall	52.6 %	53.7 %	54.4 %	55.9 %	56.2 %	56.7 %
Median relative gap size	10.4 %	5.4 %	3.1 %	2.1 %	1.7 %	1.3 %
Instances solved to optimality	6.1 %	9.4 %	11.9 %	16.0 %	16.8 %	17.0 %

**Figure 2:**
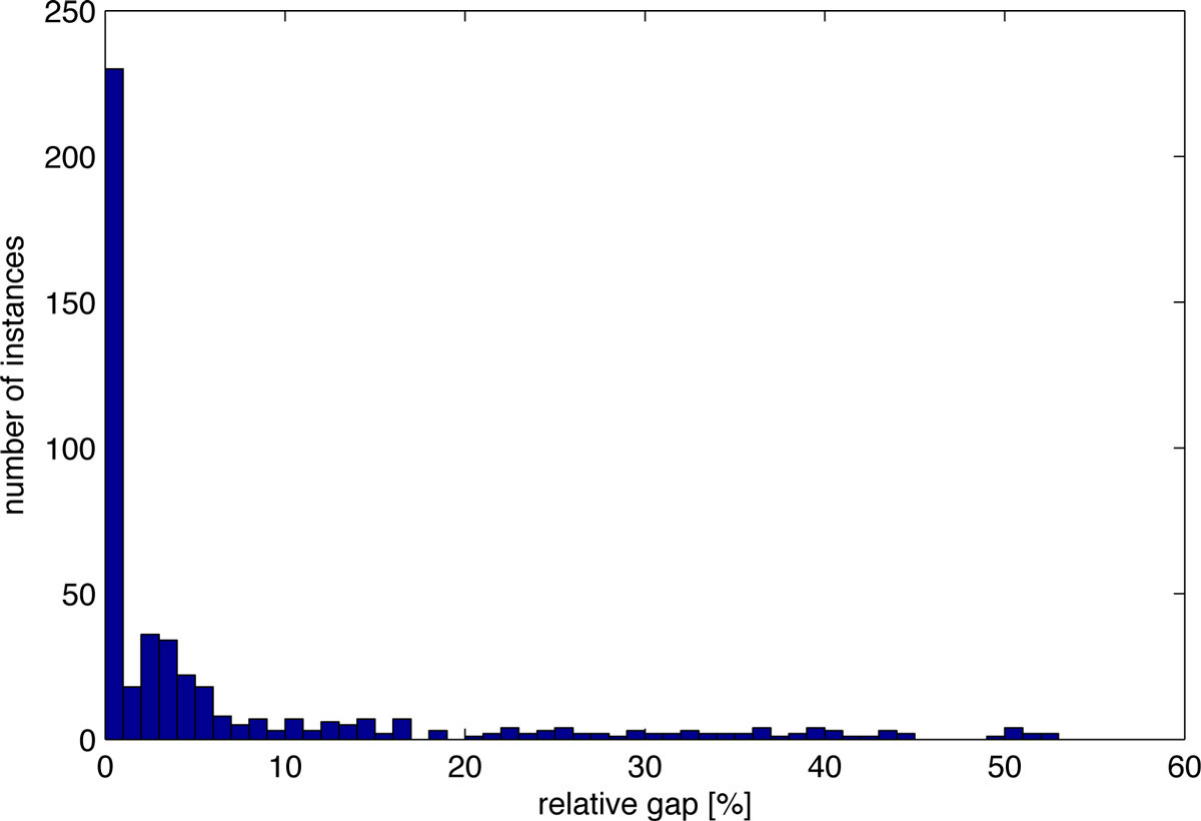
**Distribution of the relative gap in percent for the 488 instances**.

When setting the maximum runtime to 5 min, all 488 instances were solved in a total runtime of 30.2 h, out of which 78 instances were solved to optimality (16.0 %). The median relative gap was 2.1 %, which indicates that our method is able to identify matchings with a likelihood close to the maximum likelihood in many cases. Figure 2 displays a histogram of the observed relative gap. For most instances it is small, but for a few instances it constitutes more than 50 % of the likelihood of the returned solution.

### Scoring function assessment

**Figure 3:**
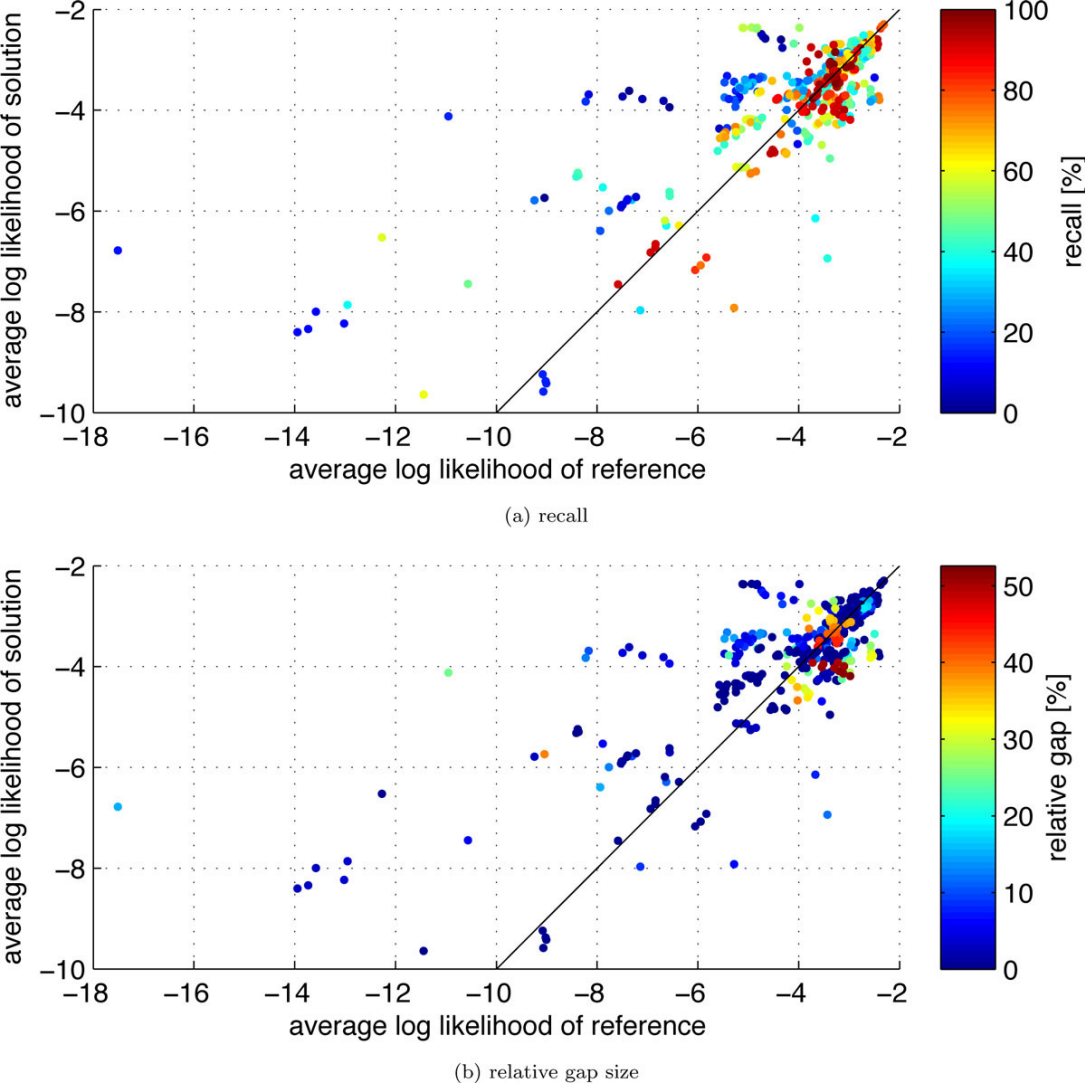
**The plots show the quality of the scoring function as measured by the average log likelihood of a unit of coevolution in our solutions versus the average log likelihood of a unit of coevolution in the reference matchings**. Points are colored according to (a) recall and (b) relative gap size.

Using the proven near-optimality of most of our solutions, we can assess the scoring function that we introduced in the Mathematical Model section. We relate the log likelihood of the reference matching to the log likelihood of our computed matching. To this end, we normalize the log likelihood of a matching such that it corresponds to the average log likelihood of a unit of coevolution. The results are displayed in Figure 3.

For instances below the bisecting line, our matching has smaller average log likelihood than the reference matching. For 64 out of the 488 instances, this applies with a difference in log likelihood of more than 0.5. This can have two reasons. First, CUPID might fail to compute a good matching, which is possible if the gap is large. Indeed, 27 out of these 64 instances have a relative gap larger than 20 %, see Figure 3b. The second reason for a reference log likelihood larger than our solution's log likelihood lies in different cardinalities of the reference matching and our solution. In these instances, a smaller matching size leads to a larger average log likelihood. Since CUPID determines maximum cardinality matchings, it cannot obtain an average log likelihood as large as the one of the reference matching, even if it solves an instance to optimality. The performance on these instances can only be improved by allowing for smaller matchings.

Instances for which the average log likelihood of our solution is larger than the average log likelihood of the reference matching are located above the bisecting line in Figure 3. For 127 out of the 488 instances, this applies with a difference in log likelihood of more than 0.5. These are instances for which the reference matching is not the matching with the highest likelihood according to the data. This can have two reasons. First, our maximum likelihood model might need to be refined. Second, the data, i.e. the multiple alignments, might be insufficient or not accurate enough to distinguish a correct from an incorrect matching. We consider the latter issue to be the more significant one as obtaining multiple alignments that accurately reflect evolutionary history is a difficult problem.

Instances close to the bisecting line are favorable instances for our scoring and algorithm. There the solution and reference matchings have similar log likelihood. In total, for 297 of the 488 instances, the difference between these two log likelihoods is at most 0.5. These are the instances for which we indeed obtain a large recall as indicated in Figure 3a by the accumulation of red points near the bisecting line. In fact, these 297 instances have an average recall of 62.5 % while it is 46.3 % for the remaining instances, which is a significant difference (*p *< 10^-10 ^according to a Wilcoxon test).

## Conclusions and discussion

In this article, we introduce a novel approach for predicting a matching of proteins in the presence of paralogs given multiple sequence alignments of two protein families. Our line of reasoning is centered around *units of coevolution*, which we identify as the minimal units of evidence for coevolution. Several properties distinguish our approach CUPID from previous ones. First, we employ a generative statistical model and score putative matchings based on their likelihood. Second, we make use of a close connection to the network alignment problem to compute provably near-maximum or maximum likelihood matchings. We observe a median relative tightness of these bounds as small as 2.1% while limiting the runtime to at most 5 minutes per instance. Third, on a commonly-used benchmark data set, CUPID performs better than three state-of-the-art methods in terms of recall and precision.

Bounds on the optimal score facilitate drawing conclusions on the quality of the scoring function. We can attribute false predictions to weaknesses of the scoring function, while for heuristic methods they could also be caused by a failure to find a good, high-scoring solution.

Our analysis shows that for many instances a matching that does not have maximum cardinality will likely result in a larger average log likelihood for a unit of coevolution. Further, reference matchings usually do not have maximum cardinality. Recall and especially precision of the predicted matching can thus be improved by allowing matchings of smaller cardinality. This could be addressed, for example, by introducing constraints into our optimization scheme that influence the matching size. Subsequently, one could apply model selection approaches to predict the size of the true matching.

So far, we have restricted ourselves to the quantities ∆*_A_*(*a, a*') and *ℓ_A_*(*a, a*') to assess sequence identity, as done previously. The corresponding scoring model is very simple and depends greatly on the quality of the underlying multiple sequence alignment, which is error-prone. We therefore consider exploring the effect of using different alignment methods and other, more fine-grained, scoring models as an interesting topic for future research. For example, we expect that results improve if alignment features such as secondary structure, amino acid substitution type or alignment confidence (using e.g. the head-or-tails [16] or GUIDANCE score [17]) are quantified and considered during the mapping. By doing so, relatively well-conserved alignment regions that are likely to participate in an interaction that is shared family-wide are upweighted. Using our current model, we could straightforwardly use only selected alignment columns for scoring a unit of coevolution, for example those with alignment confidence higher than a threshold. In order to weigh alignment columns, the scoring model would need to be revised.

Inspired by a discussion in Tillier et al. [9], another possible extension is to allow many-to-many instead of only one-to-one mappings. The scoring based on units of coevolution could immediately be adapted to such a situation. However, adapting the Lagrangian relaxation approach is less straightforward and requires more effort.

As a closing remark, we recall that mapping paralogs is only a small ingredient to the successful prediction of protein-protein interaction networks, which remains a challenging and interesting field of research.

## Competing interests

The authors declare that they have no competing interests.

## Authors' contributions

MEK, TM, IW, AS and GWK conceived and developed the method and designed the experiments. MEK implemented CUPID. MEK, TM, IW and MP carried out and analyzed the experiments. AS and GWK guided the research. All authors drafted, read and approved the final manuscript.

## Declarations

Publication of this article was funded by Centrum Wiskunde & Informatica.

This article has been published as part of BMC Bioinformatics Volume 14 Supplement 15, 2013: Proceedings of the Eleventh Annual Research in Computational Molecular Biology (RECOMB) Satellite Workshop on Comparative Genomics. The full contents of the supplement are available online at http://www.biomedcentral.com/bmcbioinformatics/supplements/14/S15.
